# CELF2 Promotes Tau Exon 10 Inclusion via Hinge Domain-Mediated Nuclear Condensation, Driving Cognitive Dysfunction in Tauopathy Models

**DOI:** 10.21203/rs.3.rs-8555050/v1

**Published:** 2026-01-19

**Authors:** Lizhen Chen

**Affiliations:** UT Health San Antonio

**Keywords:** CELF2, alternative splicing, condensate, hinge domain, intrinsically disordered region, tau, NOVA2, SFPQ, TurboID

## Abstract

Alternative splicing is a fundamental mechanism underlying protein diversity. The microtubule-associated protein tau (MAPT) undergoes age-associated alternative splicing of exon 10 to generate 3R and 4R isoforms, and disruption of the 4R:3R ratio is a central feature of tauopathies. However, the molecular mechanisms regulating tau exon 10 splicing remain incompletely understood. Here, we identify the RNA-binding protein CELF2 as a key promoter of tau exon 10 inclusion. Loss of CELF2 in the mouse brain reduces exon 10 inclusion, resulting in a decreased 4R:3R ratio. We show that an intrinsically disordered region (IDR) within the CELF2 hinge domain drives protein condensation and is essential for its splicing activity. This IDR can be functionally substituted by those of FUS or TAF15. CRISPR-based imaging reveals colocalization of CELF2 condensates with tau RNA. Proteomic analyses identify NOVA2 and SFPQ as CELF2 interactors, which co-condense with CELF2 to cooperatively regulate tau exon 10 splicing. A conserved negatively charged residue (D388) within the IDR is critical for condensate formation, protein interactions, and splicing function. Finally, CELF2 condensation capacity correlates with 4R tau expression in vivo and influences locomotor and cognitive performance. These findings uncover a condensate-based mechanism for tau splicing regulation with implications for tau-related neurodegeneration.

## INTRODUCTION

Alternative splicing (AS) plays an essential role in transcriptional regulation and is found in approximately 95% of human protein-coding genes. AS gives rise to multiple mature mRNAs encoding protein isoforms with distinct functions, structures, stability, or cellular localization across different tissues, developmental stages, or under specific conditions ^1^. The existence of distinct splice isoforms suggests unique temporal and spatial functionalities, thereby implicating AS in fundamental biological processes as well as disease pathogenesis ^2^. AS of pre-mRNA is carried out by the spliceosome, a macromolecular complex comprising small nuclear RNAs and small nuclear ribonucleoproteins (snRNPs). Within the regulatory framework, AS-related RNA-binding proteins (RBPs) play an instrumental role in regulating AS events. Sequence-specific RBPs binds to pre-mRNA to form ribonucleoprotein complexes to control AS, and each AS event is controlled by multiple RBPs.

To achieve precise spatiotemporal control over intricate biochemical reactions, cells must orchestrate the arrangement of proteins and other large molecules within subcellular regions. Beyond traditional membrane-bound organelles like the endoplasmic reticulum and Golgi apparatus, cells harbor diverse membraneless compartments. These include nucleoli, Cajal bodies, processing bodies and stress granules ^3-5^. Recent research suggests that the formation of these membraneless compartments is often mediated by a physical phenomenon called phase separation (PS). And PS has emerged as a mechanism underlying spatiotemporal protein recruitment involved in various physiological processes, including splicing regulation, transcription, signal transduction, and DNA damage repair ^6-10^. Among PS proteins, many have intrinsically disordered regions (IDRs) that lack a specific three-dimensional structure and typically are enriched with charged amino acids, polar amino acids, and/or aromatic amino acids. Multivalent electrostatic, cation-pi, pi-pi, and hydrophobic interactions have all been proposed to contribute to IDR PS ^11-17^.

RBPs are generally characterized by the RNA-recognition motifs (RRMs) and IDRs, which allow RBPs to assemble with RNAs and proteins to form dynamic phase-separated condensates. RBPs are critical players in gene regulation and are at center stage in our understanding of cellular function in both normal and disease processes. Dysfunction of RBPs and the subsequent disruption of RNA processing are increasingly implicated in neurological disorders including age-associated neurodegeneration ^18^. CELF genes encode a conserved family of RBPs that is involved in regulating gene expression at multiple levels, including alternative splicing, RNA transport, translation and mRNA stability ^19^. All 6 human CELF genes are expressed in both developing and adult brains, and the expression of CELF genes in the nervous system is evolutionarily conserved ^20-25^. CELF2, like the other five members of its family, possesses three RNA recognition motifs (RRMs) and intrinsically disordered regions (IDRs), and is known to regulate alternative splicing ^26, 27^. SNPs of the CELF2 gene have been found to be associated with late onset Alzheimer’s disease (AD) ^28, 29^, offering valuable first insight into the genetic connection. However, the mechanistic role that CELF2 may play in AD is largely unknown.

A prominent pathological feature in AD and multiple neurodegenerative diseases is the aberrant aggregation of the microtubule associated protein tau (MAPT). Through AS, MAPT gene generates six protein isoforms that differ by the presence or absence of two N-terminal domains (N1 and N2) and the second microtubule binding repeat (R2) ^30^. The inclusion or exclusion of exon 10 that encodes R2 produces 4-repeat (4R) and 3-repeat (3R) tau respectively. The major function of tau is to promote microtubule assembly and stabilize microtubules by binding to microtubules ^31, 32^. The 4R tau can promote microtubule polymerization faster than 3R tau ^30^. In the healthy adult human brain, the 4R and 3R isoforms are found approximately 1:1 ratio ^30, 33^. Alterations in the 4R:3R tau isoform ratio, often caused by mutations within exon 10 or mutations affecting exon 10 inclusion, lead to pathogenesis of various tauopathies ^34^. The significance of preserving the equilibrium between 3R and 4R isoforms in healthy neurons is underscored by the link between perturbations in this ratio and disease.

In this study, we demonstrate that CELF2 binds to MAPT mRNAs and promotes exon 10 inclusion and that loss of CELF2 in the mouse brain reduces the 4R:3R tau ratio. We further show that the CELF2 hinge domain functions as an intrinsically disordered region (IDR) to mediate phase-separated condensation. The CELF2 IDR is essential for its ability to promote tau exon 10 inclusion and can be functionally replaced by the IDRs of FUS or TAF15. Using CRISPR-based imaging, we revealed that CELF2 condensates colocalized with tau RNA. TurboID identified CELF2-interacting proteins, including NOVA2 and SFPQ, which cooperate with CELF2 to regulate tau exon 10 splicing. A conserved negatively charged residue, D388, within the IDR is critical for CELF2 condensation and function. Finally, we show that CELF2 elevates 4R tau expression *in vivo*, resulting in locomotor and cognitive deficits. Together, our findings suggest that CELF2 regulates tau alternative splicing by forming condensates through its IDR in concert with other splicing factors, and that the composition of these condensates determines tau isoform balance and neuronal function.

## RESULTS

### CLIP-seq identified MAPT(tau) as a target of CELF2 and Celf2 knockout alters 4R:3R tau ratio in mouse brain.

**Figure 1:**
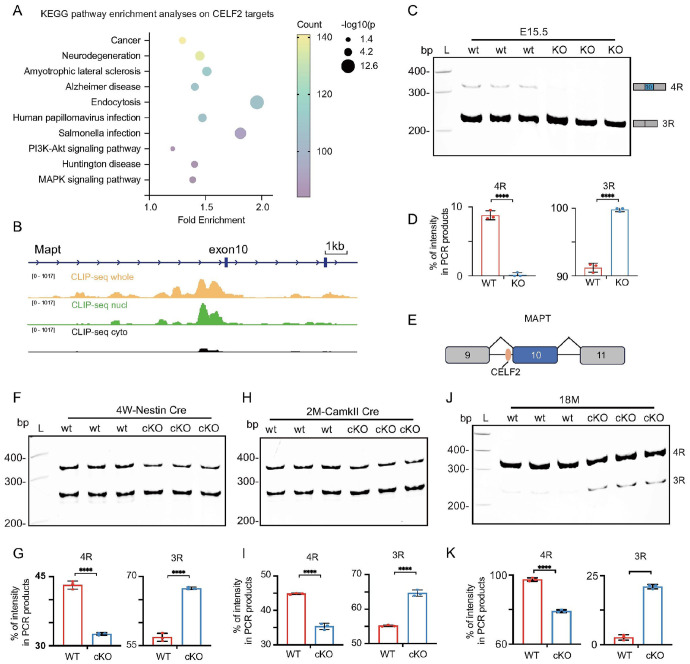
CLIP-seq identified MAPT as a target of CELF2 and Celf2 knockout reduces 4R:3R tau ratio in mouse brain. **(A)** KEGG pathway enrichment analysis on CELF2 target genes. CELF2 targets were identified from our previous CLIP-seq using N2A cells. The top 10 pathways are presented. **(B)**Genome browser view of CLIP-seq peaks on the Mapt gene locus. CELF2 specifically binds to the intron 5’-to the alternatively spliced exon 10. **(C)**RT-PCR showing that Celf2 KO eliminates 4R tau expression. Brain tissues from Celf2 KO and wildtype E15.5 mouse embryos were used to generate cDNA for RT-PCR to detect the alternative splicing of *Mapt* exon 10. **(D)**quantification of RT-PCR results to show that Celf2 KO leads to a loss of 4R tau expression in E15.5 mouse brain. n=3, *p < 0.05; ***p < 0.001; ****p < 0.0001. **(E)** A schematic model to indicate the binding of CELF2 on the intron 5’-to exon 10 to promote exon 10 inclusion. **(F-K)**RT-PCR and quantification of 4R and 3R tau expression in mouse brains at indicated stages (4 weeks, 2 months and 18 months) in Celf2 cKO induced by Nestin-Cre (F-G) or CamKII-Cre (H-K). n=3, *p < 0.05; ***p < 0.001; ****p < 0.0001.

To investigate CELF2 function, we have previously performed CLIP-seq in N2A cells to identify CELF2 targets ^35^. Different cell fractions (whole cell, cytoplasmic and nuclear) were used in CLIP-seq to distinguish CELF2 function in the nucleus and cytoplasm. The resulting 4279 nuclear target genes underwent KEGG pathway enrichment analysis, highlighting the enrichment of 108 genes linked to Alzheimer's disease (AD), including tau (Fig. 1A, 1B). CELF2 specifically binds to the intron 5’ to the alternatively spiced exon 10 (Fig. 1B). This finding aligns with previous GWAS studies that have connected CELF2 with AD ^28, 29^. We found that cytoplasmic CELF2 binds to the 3’UTR of mRNA, while nuclear CELF2 binds to introns, consistent with its nuclear function in alternative splicing (**Fig. S1A**). To confirm the regulation of tau splicing by CELF2 in brain, we examined tau exon 10 splicing in embryonic brain of Celf2 knock out (KO) mice (**Fig. S1B**) ^35^. As previously reported ^36, 37^, embryonic mouse brain expresses a low level of 4R tau, with 3R tau as the predominant tau isoform (Fig. 1C, 1D). Notably, the 4R tau was undetectable in *Celf2* KO embryonic brain (Fig. 1C, 1D). Therefore, these data indicate that CELF2 binds to the intron 5’ to exon 10 and promote axon 10 inclusion (Fig. 1E).

4R tau expression is limited in both human and rodent fetal brains ^36, 37^. As postnatal development progresses, the ratio of 3R to 4R tau isoforms in the healthy human brain transitions to a 1:1 ratio ^38^. The expression patterns of tau isoforms in adult brains vary between humans and rodents. In the adult rodent brain, the presence of 3R tau gradually reduces, with 4R tau emerging as the predominant isoform in later stages of adulthood ^36, 37^. To understand whether CELF2 regulates tau exon 10 splicing in adult brain, we crossed our previously generated Celf2 cKO allele with nestin-Cre or CamKII-Cre line and induced *Celf2* depletion at postnatal stages, as constitutive *Celf2* KO animals die at newborn ^35^. We confirmed the depletion of CELF2 in the *Celf2* cKO brain (**Fig. S1C**) and examined tau exon 10 expression at 4 weeks, 2 months and 18 months old. Unlike the embryonic brain, the young adult wildtype control brains (4weeks and 2 months old) expressed approximately equal amount of 4R and 3R tau isoforms (Fig. 1F-I). Notably, in 18 months old brains, the 3R tau was barely detectable (Fig. 1J, 1K). In all stages we examined, we found that CELF2 depletion significantly reduced 4R and increased 3R tau expression (Fig. 1F-K), indicating that CELF2 functions to promote tau exon 10 inclusion in the brain across developmental stages.

### CELF2 forms phase-separated condensates through its intrinsically disordered hinge domain

**Figure 2:**
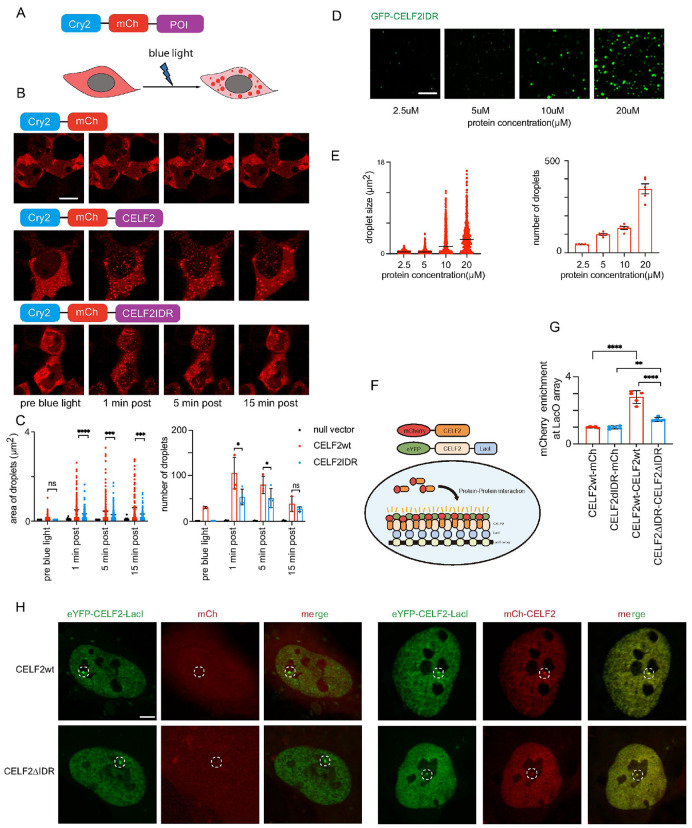
CELF2 forms phase-separated condensates through its intrinsically disordered hinge domain **(A)** Schematic diagram of the optogenetic platform to study phase separation. The construct consists of the CRY2PHR domain, mCherry fluorescent protein and the protein of interest (POI). Upon 488nm blue light activation, CRY2 will rapidly cluster, resulting in condensation of proteins with phase separation capacity. **(B)** Representative images of 293T cells transiently transfected with CRY2-mCh control, CRY2-mCh-CELF2, or CRY2-mCh-CELF2IDR constructs before and after blue light illumination. CRY2-mCh-CELF2 and CRY2-mCh-CELF2IDR formed optoDroplets, suggesting phase separation capacity of CELF2 IDR. Scale bar: 5 μm. **(C)** quantification of optodroplets. Area and number of optodroplets in 3 representative cells were measured using FIJI. Statistical analysis: one-way ANOVA, *p < 0.05; ***p < 0.001; ****p < 0.0001. **(D)**Representative images of droplets formed by recombinant GFP-CELF2IDR in the droplet formation buffer containing 10% PEG-8000 at indicated protein concentrations. **(E)**quantification of GFP-CELF2IDR droplets. Droplet size and number was measured using FIJI. **(F)** Schematic illustration of the LacO array system to test CELF2 self-interactions. eYFP-CELF2-LacI is recruited to LacO array through protein-DNA binding and mCherry-CELF2 can be recruited to the LacO array through CELF2-CELF2 interactions. **(G-H)** quantification and representative images of mCherry-CELF2 recruitment to the LacO hub through CELF2-CELF2 self-association. mCherry-CELF2 signal is enriched at the LacO hub through interacting with eYFP-CELF2-LacI, while reduced or no mCherry enrichment was detected at the eYFP hub in the CELF2ΔIDR or mCherry control groups. Enrichment of mCherry above relative level of 1 suggests protein-protein interaction. Statistics: one-way ANOVA, **P < 0.01; ****P < 0.0001. Scale bar: 10 μm.

Formation of biomolecular condensates through phase separation (PS) has emerged as a ubiquitous mechanism to promote compartmentalization for dynamic biological processes ^39-41^. Intrinsically disorder regions (IDRs) are often found in proteins that undergo PS to form condensates. Like other CELF family members, CELF2 contains an intrinsically disordered hinge domain (**Fig. S2A-C**). This prompted us to test if CELF2 undergoes PS through its IDR. We first used the optogenetic system, optoDroplet, which is based on *Arabidopsis thaliana* CRY2 ^42^. We expressed CELF2 fused to mCherry and the photolyase domain of the CRY2 protein in 293T cells (Fig. 2A). Upon blue light illumination, CRY2 undergoes self-association, leading to an increase of local concentration of the fused protein ^43^. Consistent with previous reports, CRY2-mCherry alone showed little clustering upon blue light activation. As expected for IDRs, fusing full length CELF2 or CELF2 IDR to CRY2 led to blue-light dependent clusters formation (Fig. 2B, 2C), suggesting that CELF2 IDR (the hinge domain) can drive phase-separated condensate formation. We next purified recombinant GFP-CELF2IDR (**Fig. S2D**) and performed *in vitro* droplet formation assay. Purified GFP-CELF2IDR fusion protein formed droplets in the presence of PEG8000 and the droplet size and number correlated with protein concentration (Fig. 2D, 2E), again suggesting that CELF2 IDR can drive condensates formation.

We further examined CELF2 condensation/self-interaction using a previously established imaging-based approach^44-47^. Through specific LacI-LacO interaction, eYFP-LacI-tagged protein is recruited to the LacO array, generating a concentrated interaction hub. mCherry-tagged protein is brought to the hub through multivalent interactions and the intensity of mCherry at the eYFP hub can be measured to quantify the potency of these multivalent interactions (Fig. 2F). We co-expressed mCherry-CELF2 and eYFP-CELF2-LacI in U2OS cells containing a synthetic Lac operator (LacO) array integrated to the genome ^48^. We observed a strong mCherry signal at the hub (Fig. 2G-H), indicating a strong self-interaction ability of CELF2. When we performed the assay using IDR-truncated CELF2 (ΔIDR), we observed a much weaker mCherry signal at the array, even though the recruitment of eYFP-CELF2ΔIDR-LacI molecules to LacO array appeared normal (Fig. 2G-H). Therefore, these data suggest that CELF2 IDR is required for CELF2 condensation/self-interaction.

### CELF2 condensation is critical for its function in promoting tau exon 10 inclusion

**Figure 3:**
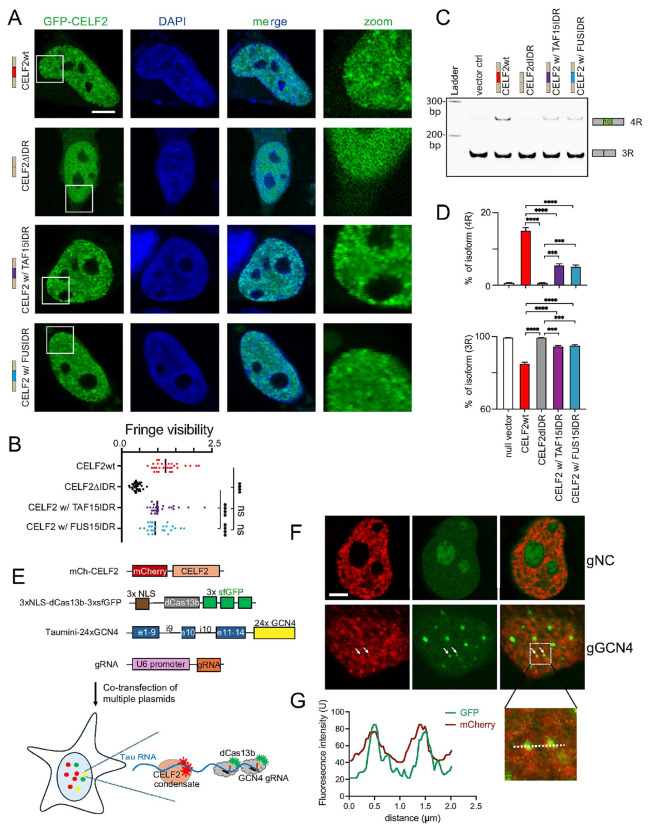
CELF2’s condensation capacity is critical for its function and CELF2 condensates colocalize with tau mRNA. **(A)**Representative confocal images of SH-SY5Y cells transiently transfected with the indicated CELF2 constructs. Deletion of CELF2IDR abolished CELF2 condensation, but replacing CELF2IDR with FUSIDR or TAF15IDR retained condensate formation capacity of CELF2. Scale bar: 5 μm. **(B)**quantifications of fringe visibility of CELF2 condensates. Fringe visibility values of randomly selected CELF2 foci from 6 cells (5 foci/cell) were plotted for each condition. Statistics: one-way ANOVA, ****P < 0.0001. **(C)**Representative gel image of RT-PCR results showing 4R and 3R tau level in SH-SY5Y cells transfected with the indicated constructs. Expression of CELF2wt but not CELF2ΔIDR promoted 4R tau expression, and CELF2IDR could be functionally replaced by the IDR of FUS or TAF15. **(D)**quantification of RT-PCR results from (C). The intensity of 4R and 3R *MAPT*bands were measured using FIJI and the percentage of each band was calculated and plotted. Statistics: one-way ANOVA, ***p < 0.001; ****p < 0.0001. **(E)**Schematic illustration of the experimental design using CRISPR-based RNA imaging to show colocalization of CELF2 condensates and tau mRNA. **(F)**Representative confocal images of 293T cells co-transfected with the plasmids indicated in (E). GFP-labeled tau RNA foci were only detected in cells co-transfected with GCN4 gRNA (bottom panels) but not in those co-transfected with the negative control gRNA (gNC) (top panels). **(G)**Line scan to show the colocalization of GFP foci (tau mRNA) and mCherry foci (CELF2 condensates).

To understand the importance of CELF2 condensation on its function, we transfected GFP-tagged CELF2wt or CELF2ΔIDR in SH-SY5Y neuroblastoma cells and examined condensate formation and tau splicing. We observed that GFP-CELF2wt was localized to the nucleus and displayed a punctate distribution pattern (Fig. 3A), suggesting that CELF2 condensation might be associated with its function in the nucleus. In contrast, GFP-CELF2ΔIDR showed a homogeneous expression pattern in the nucleus, as reflected by the low fringe visibility measuring the GFP signals (Fig. 3A, 3B), indicating that the disordered hinged domain is required for CELF2 condensate formation. When we examined tau splicing, we found that SH-SY5Y cells transfected with the vector control expressed predominantly 3R tau. In cells transfected with GFP-CELF2wt, 4R tau expression was significantly enhanced (Fig. 3C, 3D), consistent with the data obtained from the Celf2 KO mouse brain showing that CELF2 functions to promote tau exon 10 inclusion. Unlike CELF2wt, CELF2ΔIDR failed to enhance 4R tau expression (Fig. 3C, 3D), indicating that CELF2 IDR is critical for its function in regulating alternative splicing.

It has been previous shown that in some cases one IDR can be functionally replaced by another in RNP granule assembly ^49, 50^. We then asked whether CELF2 IDR could be substituted by other IDRs to regulate CELF2 condensate formation and function. Fused in sarcoma/translocated in liposarcoma (FUS/TLS or FUS) and TAF15 represent two of the most studied examples of proteins that undergo multivalent interactions and phase separation ^51^. The prion-like domain (PrLD) in FUS protein is an IDR enriched with tyrosine residues and can undergo phase separation driven by tyrosine-tyrosine interactions ^12^. TAF15 IDR has the same number of tyrosine residues but more charged residues than FUS IDR and exhibits a strong tendency to phase separate ^52^. We proceeded to replace CELF2's IDR with IDRs from FUS and TAF15, inserted between RRM2 and RRM3 (**Fig. S3A, B**). Notably, the fusion proteins (CELF2ΔIDR with FUS or TAF15 IDR) display similar condensates in cells as CELF2wt (Fig. 3A, 3B). Furthermore, CELF2 with replaced IDR was sufficient to promote 4R tau expression (Fig. 3C, 3D). These results indicate that CELF2 IDR can be functionally substituted by selective IDRs. The correlation of condensate formation status and function in promoting 4R tau expression further support that the CELF2 condensation is critical for its function.

To further elucidate the functional importance of CELF2 condensation in regulating tau splicing, we investigated whether CELF2 condensates colocalize with tau RNA. We employed the CRISPR-dCas13 RNA labeling which has been shown to be compatible with protein visualization and has high labeling efficiency ^53^. To visualize tau RNA, we generated a tau minigene plasmid with 24x GCN4 repeats (Fig. 3E) and co-transfected the plasmid with NLS-dCas13b-GFP, gRNA targeting GCN4 repeats, and mCherry-CELF2 plasmids to 293T cells. We observed GFP foci in cells transfected with gGCN4 but not in cells with negative control gRNA (gNC) (Fig. 3F). Although not all GFP-labeled tau RNA foci co-localized with mCherry-CELF2 condensates, a subset showed clear colocalization (Fig. 3G), supporting the role of CELF2 condensates in regulating tau splicing.

### CELF2 forms heterotypic condensates with NOVA2 and SFPQ through its IDR

**Figure 4:**
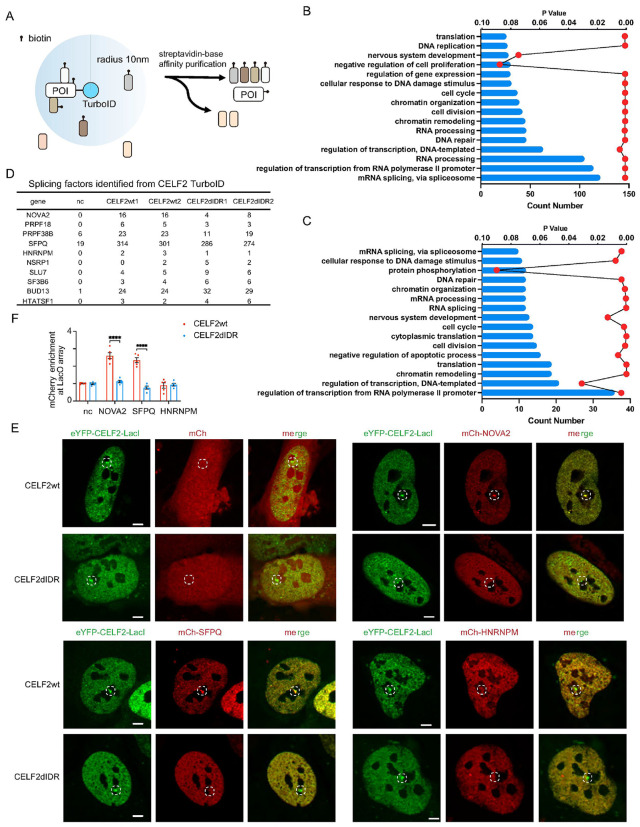
TurboID identified CELF2 cofactors with which CELF2 forms heterotypic condensates through its IDR **(A)**Schematic illustration of proximity-dependent biotin identification technique, TurboID, to identify cofactors of CELF2wt and CELF2ΔIDR. **(B)** KEGG pathway enrichment analysis on turboID-identified CELF2 cofactors. Top 16 pathways ranked by Count are plotted. mRNA splicing pathway is among the most significantly enriched pathways. **(C)** KEGG pathway enrichment analysis on turboID-identified CELF2 cofactors that show differential interaction with CELF2wt vs CELF2ΔIDR. Top 16 pathways ranked by Count are plotted. **(D)**Peptide numbers of the splicing factors identified from Turbo ID using empty vector, CELF2wt or CELF2ΔIDR as baits. Peptide numbers of two independent experiments are shown. NOVA2 and SFPQ showed reduced peptide numbers in CELF2ΔIDR than in CELF2wt TurboID, and were then selected for further experiments to test their interactions with CELF2. **(E)**Representative images of mCh-NOVA2, mCh-SFPQ and mCh-HNRNPM recruitment to the LacO hub through CELF2-cofactor interactions in U2OS cells. mCh-NOVA2 and mCh-SFPQ, but not mCh-HNRNPM, were enriched at the LacO hub, and the enrichment was dependent on CELF2 IDR. Scale bar: 5 μm **(F)**quantification of the multivalent interactions between CELF2 and its cofactors. Enrichment of mCherry above relative level of 1 suggests protein-protein interaction. Statistics: one-way ANOVA, ****P < 0.0001.

Given that an alternative splicing (AS) event is regulated by multiple RNA-binding proteins (RBPs) and that IDRs facilitate both homotypic and heterotypic interactions, we sought to identify the RBPs that interact with CELF2, forming heterotypic condensates and collectively regulating AS. To capture CELF2-interacting proteins, we used the proximity-dependent biotin identification TurboID technique ^54^ with CELF2 as the bait protein (Fig. 4A). Fusing TurboID to CELF2 allowed labeling molecules interacting with CELF2. The labeled interactors were then pulled down with streptavidin peroxidase beads followed by proteomic profiling (**Fig. S4A**). Consistent with the function of CELF2 in regulating alternative splicing, the identified CELF2-interacting proteins were most significantly enriched for proteins involved in RNA splicing via spliceosome (Fig. 4B). We also performed TurboID for CELF2ΔIDR and found that IDR deletion diminished the interaction between CELF2 and a list of its interactors. KEGG pathway enrichment analysis on these interactors again identified RNA splicing-related proteins (Fig. 4C). Among the splicing-related proteins whose interaction with CELF2 was weakened by IDR deletion (Fig. 4D), we selected NOVA2 and SFPQ for further investigation due to their function in neuronal splicing and neurodegeneration ^55-57^.

We next interrogated whether CELF2 co-localized with SFPQ and NOVA2 in cells. We selected 293T cells for our study due to their low expression levels of CELF2, ensuring that the introduced expression patterns would not be confounded by endogenous CELF2 expression. When expressed individually, mCh-NOVA2 and mCh-SFPQ were localized in the nucleus, with a weakly punctate pattern (**Fig. S4B**). When co-expressed with GFP-CELF2, we observed a high level of co-localization between GFP-CELF2 and mCh-NOVA2 (or mCh-SFPQ) (**Fig. S4C, S4D**). Interestingly, in the presence of GFP-CELF2, both mCh-NOVA2 and mCh-SFPQ displayed a strongly punctate pattern as measured by the higher fringe visibility of mCherry signal (**Fig. S4C-F**). This suggests that CELF2 might facilitate to recruit NOVA2 and SFPQ to those nuclear condensates. We further found that the CELF2-dependent NOVA2 and SFPQ condensates required the IDR of CELF2, as mCh-NOVA2 and mCh-SFPQ showed significantly weaker condensation when co-expressed with CELF2ΔIDR (**Fig. S4C-F**).

To further investigate the heterotypic interactions between CELF2 and cofactors, we employed the LacO array imaging assay. We co-expressed eYFP-CELF2-LacI and mCherry-tagged cofactors in U2OS cells containing a synthetic Lac operator (LacO) array. Compared to mCherry control which did not show enrichment at the eYFP + hub, mCh-NOVA2 and mCh-SFPQ was highly enriched (Fig. 4E, 4F), suggesting a strong multivalent interaction between CELF2 and NOVA2 or SFPQ. As a negative control, we also examined HNRNPM, which was identified as a weak CELF2 interactor from TurboID (Fig. 4D), and observed no enrichment of mCh-HNRNPM on the eYFP + hub, indicating the specificity of CELF2-NOVA2 and CELF2-SFPQ interactions. Notably, these heterotypic interactions were dependent on CELF2 IDR, as IDR deletion abolished the enrichment of NOVA2 and SFPQ (Fig. 4E, 4F). Together, these data suggest that CELF2 forms heterotypic condensates with NOVA2 and SFPQ through its IDR.

### CELF2 co-regulates tau splicing with NOVA2 and SFPQ

**Figure 5:**
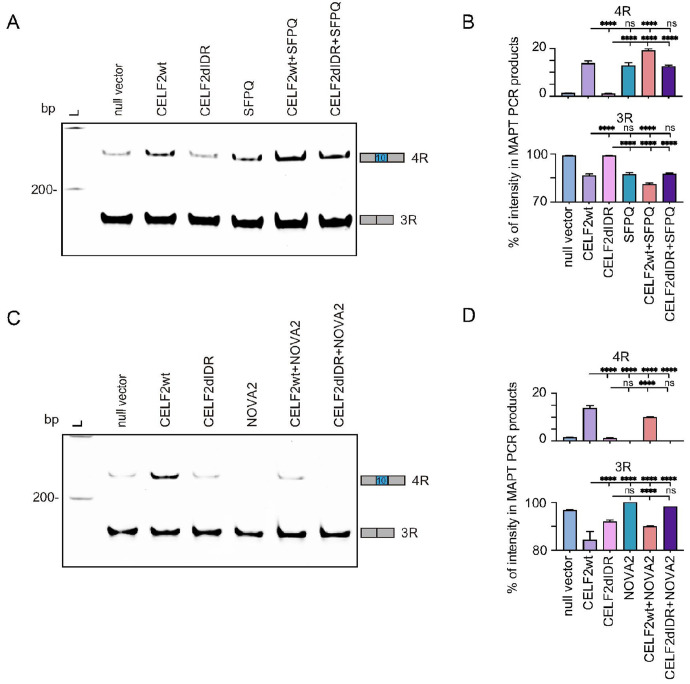
CELF2 co-regulates tau splicing with NOVA2 and SFPQ **(A)**Representative gel image showing 4R and 3R tau expression in SH-SY5Y cells transfected with . with indicated plasmid(s). Cells were transfected for 48 hours before RNA extraction and RT-PCR to examine alternative splicing of the endogenous *MAPT* gene. **(B)**quantification of RT-PCR shown in (A). Both CELF2 and SFPQ promote 4R tau expression. Co-expression CELF2 and SFPQ had a greater effect on exon 10 inclusion than either CELF2 or SFPQ expression alone. FIJI was used to measure the intensity of each *MAPT* transcript product and the percentages of 4R and 3R tau were plotted. Statistics: one-way ANOVA, ****, p < 0.0001. **(C)**Representative gel image showing the effect of CELF2 and NOVA2 on *MAPT*alternative splicing. SH-SY5Y cells were transfected with indicated plasmid(s) for 48 hours, followed by RNA extraction and RT-PCR. **(D)**quantification of RT-PCR shown in (C). CELF2 promotes exon 10 inclusion, while NOVA2 promotes exon 10 skipping. Co-expression of NOVA2 with CELF2 dampened the effect of CELF2 on promoting 4R tau expression. FIJI was used to measure the intensity of each *MAPT* transcript product and the percentage of 4R and 3R tau was plotted. Statistics: one-way ANOVA, ****p < 0.0001.

Having shown that CELF2 interacts with NOVA2 and SFPQ in nuclear condensates, we asked if they functioned together to regulate AS. Similar to CELF2, overexpression of SFPQ in SH-SY5Y cells led to enhanced 4R tau expression, although its effect was not as strong as CELF2 (Fig. 5A, 5B). Simultaneously expressing both CELF2 and SFPQ resulted in a higher 4R tau level than expressing either CELF2 or SFPQ alone (Fig. 5A, 5B), while co-expression of SFPQ and CELF2ΔIDR had a similar effect as SFPQ overexpression. Therefore, both CELF2 and SFPQ can promote tau exon 10 inclusion and they can coordinate to achieve a higher level of 4R tau expression.

Unlike CELF2 or SFPQ, NOVA2 overexpression inhibited tau exon 10 inclusion, resulting in a complete depletion of 4R tau (Fig. 5C, 5D). In addition, NOVA2 expression was sufficient to diminish CELF2-induced 4R tau expression, suggesting that NOVA2 might compete with CELF2 to promote exon 10 skipping. To further understand the function of NOVA2 in regulating tau splicing, we generated stable SH-SY5Y cell lines expressing Dox-inducibe shRNAs targeting NOVA2 (**Fig. S5A**). We noticed that 4R tau expression was barely detectable in the stable cell line. This was likely due to maturation of SH-SY5Y cells during the process of establishing stable cell lines (**Fig. S5B, S5C**). Upon Dox treatment to induce NOVA2 knockdown, 4R tau expression was significantly elevated (**Fig. S5B, S5C**), again suggesting that NOVA2 function to inhibit exon 10 inclusion. We next established stable cell lines with Dox-inducible CELF2 expression and shRNA targeting NOVA2 to test if CELF2-induced exon 10 inclusion could be elevated after knocking down NOVA2. Indeed, we found that knocking down NOVA2 in cells with CELF2 overexpression was able to further enhance 4R tau expression (**Fig. S5D, S5E**). Together, these data suggest that CELF2 interacts with other RBPs in biomolecule condensates to co-regulate AS, and that the components within the condensates determine the splicing outcomes.

### The charged residue D388 is critical for CELF2 condensate properties and CELF2 function

**Figure 6:**
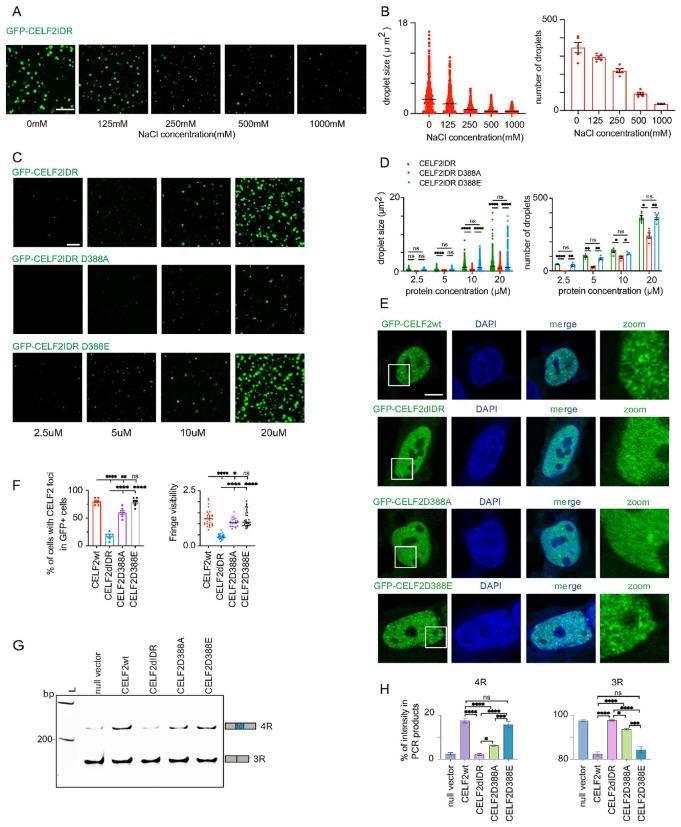
The charged residue D388 is critical for CELF2 condensate properties and CELF2 function **(A)**Representative images of *in vitro* droplets formed by recombinant CELF2 IDR in droplet formation buffers containing10% PEG-8000 and with various salt concentrations. Scale bar: 5 μm. **(B)**quantification of CELF2IDR droplet size and number at indicated conditions using FIJI. Droplet size and number negatively correlates with NaCl concentration. **(C)**Representative images of *in vitro* droplets formed by wildtype and mutant CELF2 IDRs at indicated protein concentrations. Scale bar: 5 μm. **(D)**quantification of droplet size and number of wildtype and mutant CELF2 IDRs at indicated protein concentrations. D388A mutation led to smaller and fewer droplets, while D388E mutation did not affect droplet size or number. **(E)**Representative confocal images of GFP-CELFwt, GFP-CELF2ΔIDR, GFP-CELFD388A and GFP-CELF2D388E in SH-SY5Y cells. Scale bar: 5 μm. **(F)**quantification of CELF2 condensates in SH-SY5Y cells. D388A mutation resulted in an reduction in the percentage of cells with CELF2 condensates, as well as a lower fringe visibility of GFP+ condensates. Statistics: one-way ANOVA, *p < 0.05, **p < 0.01, ****p < 0.0001. **(G)**Representative RT-PCR gel image showing 4R and 3R tau expression in SH-SY5Y cells transfected with wildtype and mutant CELF2 genes. **(H)**quantification of RT-PCR shown in (G). D388A mutation significantly reduced the effect of CELF2 in promoting 4R tau expression, while D388E did not show much impact. FIJI was used to measure the intensity of each *MAPT* transcript product and the percentages of 4R and 3R tau were plotted. Statistics: one-way ANOVA, *p < 0.05, ***p < 0.001, ****p < 0.0001.

IDRs that mediate phase separation (PS) are often enriched with charged amino acids, polar amino acids, and/or aromatic amino acids, which have all been proposed to contribute to IDR PS ^11-17^. In the *in vitro* droplet formation assay, we observed that the droplet size and number negatively correlated with the concentration of NaCl in the droplet formation buffer (Fig. 6A, 6B). Based on their overall charge composition, IDRs can exhibit either salt-out or salt-in behavior. This phenomenon is influenced by the screening of electrostatic interactions and Hofmeister effects ^58-60^. The sensitivity of IDR droplets to salt concentration suggest that charged residues might contribute to CELF2 phase separation. Within CELF2 IDR, there is only one negatively charged residue (D388) and zero aromatic residue (**Fig. S6A**). Intriguingly, this negatively charged amino acid is conserved among all 6 CELF proteins (**Fig. S6B**), suggesting its functional importance.

To investigate whether the negative charge of D388 plays a role in regulating CELF2 condensation and function, we generated two CELF2 variants: D388A to replace the negatively charged D residue with a non-charged residue A, and D388E to replace the aspartic acid with another negatively charged amino acid glutamic acid. We first Purified the IDR fragments (**Fig. S6C-E**) and performed *in vitro* droplet assay. We found that both GFP-CELF2IDR(D388A) and GFP-CELF2IDR(D388E) formed droplets. However, the D388A droplets were significantly smaller and fewer than WT when examined at the same protein concentrations (Fig. 6C, 6D). And the minimal concentration required for droplets formation was higher for to IDR(D388A), suggesting that the D to A mutation inhibits CELF2 IDR condensation propensity. Interestingly, GFP-CELF2IDR(D388E) behaved similarly as GFP-CELF2IDR(WT), forming comparable sizes and numbers of droplets at all concentrations tested (Fig. 6C, 6D).

We next determined whether D388 was required for CELF2 condensation in cells and for CELF2 function in regulating alternative splicing. In line with the results of *in vitro* droplet formation assay, GFP-CELF2(D388A) formed less distinct condensates in SH-SY5Y cells, as measured by the reduced fringe visibility (Fig. 6E, 6F). D388A also significantly reduced the number of cells with CELF2 condensates, although the effect was not as strong as ΔIDR. In contrast, D388E did not show significant changes in condensate fringe visibility or number of cells with condensates when compared to WT (Fig. 6E, 6F). Like CELF2(WT), which induced tau exon 10 inclusion upon overexpression in SH-SY5Y cells, both CELF2(D388A) and CELF2(D388E) were able to promote the expression of 4R tau. However, the expression level of 4R tau in cells expressing CELF2(D388A) was lower than that observed in cells with CELF2(WT) expression, whereas the level of 4R tau in CELF2(D388E) expressing cells was comparable to that in CELF2(WT) expressing cells (Fig. 6G, 6H). This was not due to a difference in the expression level of CELF2 variants (**Fig. S6F**). Therefore, replacing the negatively charged D388 with a non-charged residue abolished CELF2 condensation and function, while replacing D388 with another negatively charged amino acid did not affect CELF2 condensation or function.

**Figure 7:**
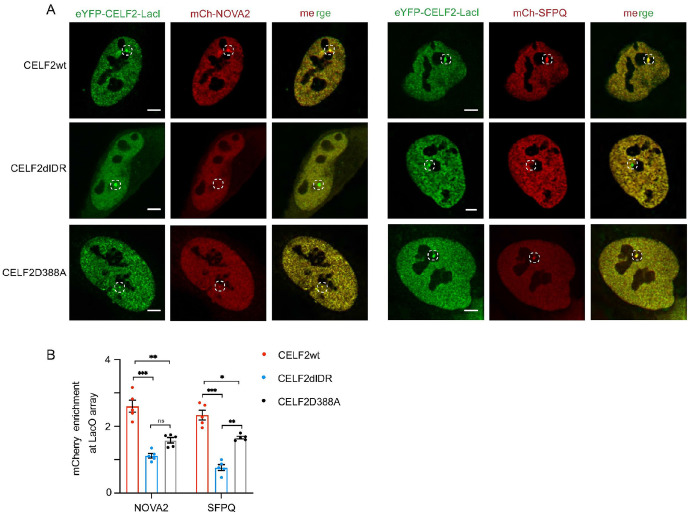
D388 within CELF2 IDR is critical for CELF2 interaction with NOVA2 and SFPQ. **(A)** Representative images of mCh-NOVA2 and mCh-SFPQ enrichment at the LacO hub. U2OS cells carrying the LacO array were co-transfected with EYFP-CELF2-LacI (*wt*, *ΔIDR*, or *D388A*) and a mCherry vector (mCh-NOVA2, mCh-SFPQ, or mCh-null vector). Scale bar: 5 μm **(B)** quantification of the multivalent interactions between CELF2 and NOVA2 (or SFPQ). Enrichment of mCherry above relative level of 1 suggests protein-protein interaction. D388A mutation partially abolished CELF2-cofactor interactions. Statistics: one-way ANOVA, ****P < 0.0001.

As CELF2 requires its IDR to interact with NOVA2 an dSFPQ (Fig. 4), we asked whether D388 influenced CELF2’s heterotypic interactions using the LacO array imaging assay. We co-expressed eYFP-CELF2 (WT or D388A)-LacI and mCh-NOVA2 (or mCh-SFPQ) in U2OS 2-6-3 cells. The enrichment of mCh-NOVA2 or mCh-SFPQ at the eYFP + hubs was significantly eliminated by D388A mutation (Fig. 7A, 7B), suggesting that the charged residue D388 is critical for CELF2 heterotypic interactions. Taken together, these data suggest that the negatively charged residue within CELF2 IDR can modulate CELF2 condensation and thus impact its function.

#### CELF2-mediated elevation of 4R tau disrupts locomotor and cognitive function in vivo

**Figure 8:**
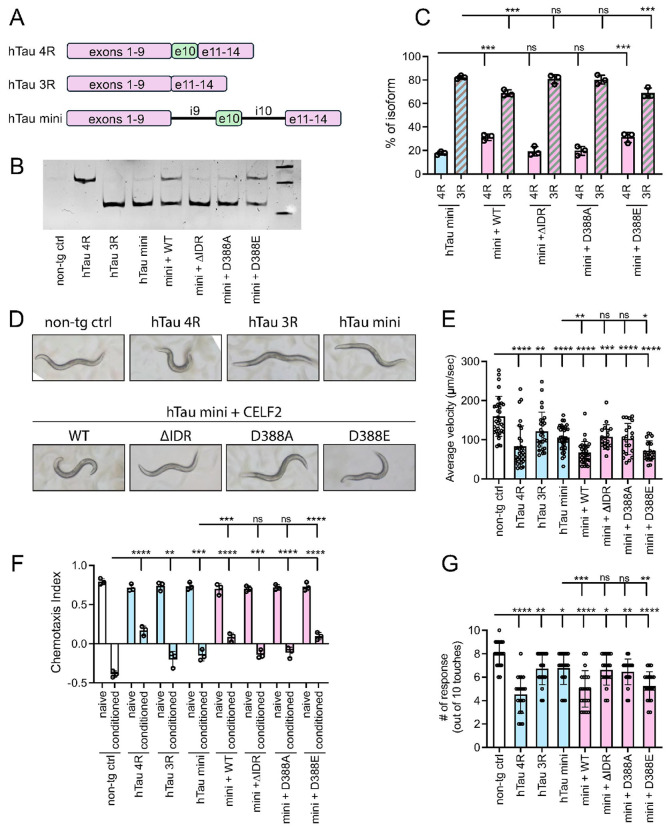
CELF2-mediated elevation of 4R tau disrupts locomotor and cognitive function *in vivo*. **(A)**Schematic illustration of human tau constructs used to generate tau transgenic *C. elegans* strains. The tau minigene construct includes mini-introns that harbor CELF2 binding sites. **(B-C)**Representative RT-PCR gel image and quantification showing 4R and 3R tau mRNA expression levels in the indicated transgenic strains. Transgenic expression of tau minigene alone produced predominantly 3R tau. Co-expression of wildtype CELF2, but not condensation-deficient CELF2 mutants (ΔIDR and D388A) enhanced 4R tau expression. **(D)**Representative images of Day-1 young adult *C. elegans* of indicated genotypes. Transgenic animals with 4R tau overexpression and those with tau minigene and CELF2 (WT or D388E) co-expression displayed an Unc phenotype with a shorter body length. **(E)**quantification of crawling speed of Day-1 young adult *C. elegans* of indicated genotypes. **(F)**quantification of chemotaxis index of the indicated genotypes and conditions in chemotaxis assay for evaluation of memory-like behavior. Expression of human tau in *C. elegans* neurons impaired memory. Co-expression of hTaumini with CELF2WT, but not ΔIDR or D388A mutants, showed more severe deficits than expression of Tau mini alone. **(G)**quantification of touch response of Day-1 young adult *C. elegans* of indicated genotypes. Statistics: one-way ANOVA, *P < 0.05, **P < 0.01, ***P < 0.001, ****P < 0.0001.

Imbalance in 4R/3R tau ratio has been indicated to disrupt cytoskeletal dynamics and promote tau aggregation, contributing to neurodegenerative tauopathies ^61^. Having shown that CELF2 condensation is critical for its role in regulating tau splicing *in vitro*, we next investigated whether CELF2 condensation regulates tau splicing and neuronal function *in vivo* using *C. elegans* as a model. We generated a series of transgenic strains expressing human tau with and without human CELF2 and subsequently examined tau isoform expression and animal phenotypes (Fig. 8). Transgenic expression of the tau minigene under a pan-neuronal promoter resulted in a predominant 3R tau expression (Fig. 8A-C). Co-expression of wildtype human CELF2 with the tau minigene significantly increased 4R tau expression, consistent with CELF2’s role in promoting tau exon 10 inclusion. In contrast, co-expression of CELF2 condensation-deficient mutants (ΔIDR and D388A), failed to elevate 4R tau levels, whereas CELF2D388E mutant exhibited an effect comparable to wildtype CELF2 in promoting 4R tau expression (Fig. 8B-C). These results demonstrate that CELF2’s condensation capacity is critical for its ability to promote 4R tau expression *in vivo*.

Evaluating locomotion and cognitive behaviors in *C. elegans* has become a powerful approach to investigate the integrated function of the nervous system ^62^. Owing to its well-mapped neural circuitry and genetic tractability, transgenic *C. elegans* models have long served as valuable tools for elucidating the molecular and cellular mechanisms underlying neurodegeneration. We found that overexpression of 4R tau in the *C. elegans* nervous system resulted in an uncoordinated (Unc) phenotype and significantly reduced locomotion velocity, whereas overexpression of 3R tau produced milder defects (Fig. 8D-G). Expression of the tau minigene alone elicited similar phenotypes to 3R tau. Consistent with their effects on 4R tau expression, co-expression of wildtype CELF2 or CELF2D388E mutant with the tau minigene recapitulated the 4R tau overexpression phenotype, while co-expression of CELF2ΔIDR or CELF2D388A did not (Fig. 8D-G). To further assess neuronal function, we performed chemotaxis assay to evaluate the cognitive function of the transgenic animals. We detected severe function impairment in animals overexpressing 4R tau as well as those co-expressing the tau minigene and CELF2 (WT or D388E) (Fig. 8F). Similar results were observed in the gentle touch response assay (Fig. 8G). Together, these data support that the condensation capacity of CELF2 is critical for regulating tau isoform expression and neuronal function *in vivo*.

## DISCUSSION

The orchestration of every alternative splicing event depends on the concerted effort of multiple RBPs. But how different proteins are recruited to the splicing sites are not clear. Through multiple approaches, we demonstrated that CELF2 forms phase-separated concentrates with other splicing regulators through its disordered hinge domain to regulate alternative splicing. Among the CELF2 targets identified by our CLIP-seq, we selected tau to study the functional significance of CELF2 condensation and its regulation. Our *in vivo* and *in vitro* studies revealed that CELF2 acts to promote tau exon 10 inclusion. The intrinsically disordered hinge domain of CELF2 mediates CELF2 condensation and activity. Our TurboID identified CELF2-interacting proteins including NOVA2 and SFPQ, which co-condensated with CELF2 to regulate alternative splicing, with NOVA2 inhibiting but SFPQ promoting tau exon 10 inclusion. The finding that the negatively charged aspartic acid residue within the IDR (D388) is essential for both homotypic and heterotypic interactions, as well as for CELF2's function, aligns with the conservation of this critical residue among CELF paralogs. Finally, CELF2 condensation capacity correlates with 4R tau expression, which leads to locomotor and cognitive impairments *in vivo*. These data highlight the importance of the disordered hinge domain of RBPs in mediating the formation of biomolecular condensates and suggest that the makeup of proteins within the splicing condensates dictates the outcomes of alternative splicing events.

### CELF2 is involved in AD by regulating alternative splicing of Tau and other AD-related genes

Alternative splicing affects more than 95% of human genes, significantly contributing to the diversity and complexity of proteins ^63, 64^. Recent investigations into alternative splicing in aging brains have uncovered specific aberrant splicing events linked to AD ^65-67^. Moreover, genome-wide association studies (GWAS) have identified associations between CELF genes and various inherited neurological conditions ^68,69^, and specific single nucleotide polymorphisms (SNPs) in the CELF2 gene have been linked to AD ^28, 29^. While these studies provide important insights into potential genetic links, the exact role of CELF2 in the pathogenesis of Alzheimer’s disease remains unclear. Our CLIP-seq revealed that CELF2 targets are enriched with genes involved in neurodegenerative diseases. Loss of CELF2 consistently leads to a reduction in the 4R:3R ratio, even though this ratio in the mouse brain gradually changes during development. However, it remains to be investigated whether the dynamic changes in the 4R:3R ratio during development and aging are related to variations in CELF2 expression. Manipulation of this ratio has been demonstrated to induce AD-like phenotypes ^70, 71^. Therefore, CELF2 may play a crucial role in the development of AD by controlling the levels of tau isoforms. Certainly, the function of CELF2 in AD is not limited to its regulation on tau splicing, as many other AD-related genes are targets of CELF2 and CELF2 regulates many aspects of RNA metabolism and function.

### CELF2’s condensation capacity is critical for CELF2 function in regulating tau splicing

All six CELF members share a similar structure, which includes three RRMs, two located at the N-terminal and one at the C-terminal, with a divergent domain situated between RRM2 and RRM3. The divergent domain exhibits limited homology among CELF protein family members and does not show significant homology to other proteins. RRMs of CELF proteins are known to play important roles in mediating protein-RNA interactions ^19^. While the divergent domain itself may not function as an RNA-binding domain, evidence suggests it likely plays a crucial role in facilitating RNA interaction. In previous yeast three-hybrid experiments, deletions within the divergent domain had the most significant impact on CELF1 binding, surpassing alterations in the RRMs ^72, 73^. Intriguingly, studies utilizing chimeric proteins revealed that the sequence of the divergent domain associated with the first two RRMs of CELF4 strongly influences binding ^74^. However, it remains unclear whether the divergent domain affects RNA binding through mediating protein-protein interactions or by conveying essential conformational states. In this work we show that the divergent domain is sufficient to form phase-separated droplets *in vitro* and is required for CELF2 condensation in cells. Consistent with the previous report on the essential role of the divergent domain of CELF2 in regulating CFTR pre-mRNA splicing ^75^, deletion of CELF2 divergent domain leads to a significant functional deficit in regulating tau splicing (Fig. 3). Changing the conserved negatively charged residue D388 to a non-charged residue disrupted CELF2 condensation and attenuated its ability to promote 4R tau expression (Figs. 6 & 8), further supporting the importance of CELF2’s condensation capacity in its function. Interestingly, CELF2 IDR (divergent domain) can be functionally substituted by FUS and TAF15 IDRs (Fig. 3). FUS/TAF15 IDRs can self-associate to form phase-separated condensates under physiological conditions ^76^. The functional substitution indicate that the specific sequence of the divergent domain may not be critical for CELF2's function. Rather, the intrinsically disordered nature of this domain plays a pivotal role in the formation of membraneless condensates. This mechanism has been shown to be essential for recruiting various components and enhancing local concentrations to facilitate functional activities ^40^. It’s worth noting that although chimeric proteins with FUS/TAF15 IDR and CELF2ΔIDR formed condensates in cells and promoted 4R tau splicing, they were not as effective as CELF2 in enhancing 4R tau splicing. This partial restoration could be attributed to the unique biophysical characteristics of condensates formed by CELF2 IDR compared to those formed by other IDRs. This observation aligns with our previous research, which demonstrated the influence of condensate biophysical properties on protein function ^47^.

### CELF2 and other splicing factors form dynamic splicing condensates to co-regulate alternative splicing

Within the genome, the same genomic sequences often serve as binding sites for multiple RBPs. RBPs frequently collaborate in complexes during splicing, influencing each other's binding specificity and functional outcomes. This interplay among RBPs adds layers of complexity to the regulatory landscape of RNA processing and gene expression. Through turboID, we identified CELF2-interacting proteins, particularly proteins interacting with CELF2 through CELF2 IDR. Among them, we focused on NOVA2 and SFPQ due to their known function in regulating alternative splicing and association with neurodegenerative diseases. Cellular localization of these proteins suggests that CELF2 might recruit NOVA2 and SFPQ to nuclear condensates through IDR-mediated heterotypic interactions between CELF2 and cofactors (Fig. 4). Functionally, both CELF2 and SFPQ can promote tau exon 10 inclusion and they can coordinate to achieve a higher level of 4R tau expression. In contrast, NOVA2 acts to inhibit tau exon 10 inclusion and might compete with CELF2 (Fig. 5). These findings support that CELF2 interacts with other splicing regulators within biomolecule condensates, jointly influencing AS. Moreover, they suggest that the composition of these condensates determines the splicing outcomes. The balance between 4R and 3R tau isoforms in the human brain changes throughout development and aging, with 3R tau predominantly prevailing during brain development, and a gradual increase in 4R tau levels occurring, eventually reaching parity with 3R tau in adulthood ^77^. Thus, we propose that the splicing condensates undergo dynamic alterations to achieve temporally and spatially specific alternative splicing.

### The conserved negatively charged residue D388 modulates CELF2 condensation and function

A wide range of molecular forces contribute to the formation of biomolecular condensates ^11-17^. IDRs are commonly enriched with aromatic residues (phenylalanine, tryptophan and tyrosine) which mediate pi-pi interactions. Other interactions that have been suggested as important drivers of biomolecular phase separation are charge-charge interactions and Pi-cation interactions. In addition, hydrophobic amino acids, such as leucine, isoleucine, and valine, are often enriched in protein regions that drive phase separation. While hydrophobic interactions may be less predominant in the context of phase separation compared to folded proteins, they likely remain important due to the significant presence of hydrophobic amino acids ^78^. There is only 1 charged residue (D388) and no aromatic residue within the divergent domain. The negatively charged residue is conserved among the 6 CELF family members. We found that replacing D388 with an un-charged amino acid (Alanine) reduced CELF2 IDR droplet formation and tau exon 10 inclusion. D388A also diminished the interaction between CELF2 and NOVA2 or SFPQ (Fig. 7). Although the charged D388 plays an important role in modulating CELF2 condensation and function, it might not be the driving force for CELF2 IDR condensation. This is because charge-driven phase separation often occurs in IDRs containing both positively and negatively charged residues or between two oppositely charged biomolecules ^78^. Therefore, the hydrophobic amino acids in IDR might be the major driving force for CELF2 condensation given their high abundance. Our observations that mutating a single conserved residue within the CELF2 IDR is sufficient to disrupt CELF2 condensation and alter its regulation of alternative splicing, ultimately impacting locomotor and cognitive function in tauopathy models (Fig. 8), suggests that targeting CELF2 condensation might represent a potential strategy to intervene neurodegenerative diseases.

### Limitations of the study

Splicing reactions occur within the spliceosome, a dynamic RNA-protein complex. This complex exhibits remarkable plasticity in substrate recognition, allowing it to incorporate various segments of a pre-mRNA into a mature mRNA, under the influence of numerous regulatory proteins ^2^. RNAs can orchestrate the assembly of multiple RBPs to condensates and dictate condensate composition ^79^. Furthermore, RNA secondary structure can influence the physical characteristics of a condensate and, independently of proteins, facilitate the formation of gel-like phases. Moreover, RNAs can modify protein conformation to trigger phase separation in response to environmental cues ^80-82^. Our study reveals that CELF2 and other RBPs co-condensate to regulate mRNA alternative splicing. However, it remains to be investigated how mRNA impact the physical properties of CELF2 condensates.

## Materials and Methods

### Cell culture and stable cell lines

SH-SY5Y and 293T cell lines were obtained from ATCC, and were cultured in Dulbecco’s Modified Eagle’s Medium (DMEM) supplemented with 10% FBS. Both cell lines were grown in a humidified incubator with 5% CO_2_. To generate the doxycycline-inducible CELF2-overexpressing cell lines, CELF2 cDNAs were individually cloned into pCR8/GW/TOPO and then transferred to the pInducer20 destination vector (pInducer20 was a gift from Stephen Elledge; Addgene #44012) using the Gateway system (Invitrogen), followed by co-transfection with packaging plasmids (psPAX2 and pMD2.G from Addgene) into 293T cells. Culture medium containing lentivirus particles were harvested, filtered, and used to infect SY5Y cells. Cells were selected by 1000 μg/ml G418 (Invitrogen) after infection to set up doxycycline-inducible stable cell lines. To generate doxycycline inducible NOVA2 knockdown cell line, NOVA2 shRNA was sub-cloned into Tet-pLKO-puro, a gift from Dmitri Wiederschain (Addgene #21915). Stable cell lines were generated after puromycin selection. Doxycycline at a concentration of 100 ng/ml was used to achieve NOVA2 knockdown.

### RNA isolation, qRT-PCR, and isoform detection

Total RNA from cultured cells was isolated using RNeasy Mini Kit (Qiagen) and total RNA from mouse brain or *C. elegans* was isolated using trizol according to the manufacture’s protocols. 500 ng RNA was used for reverse transcription with iScript Select cDNA Synthesis Kit (Bio-Rad) in the presence of both oligo (dT) and random primers. qPCR was conducted with SsoAdvanced Universal SYBR Green Supermix (Bio-Rad) using CFX384 Real-Time PCR Detection System (Bio-Rad) according to the manufacturer’s instructions. Relative expression of RNAs was determined by the ΔΔCT method using GAPDH as an internal control for quantification analyses of gene targets. To detect the 4R and 3R tau isoforms, PCR were performed with primers recognizing exon9 and exon11, followed by gel electrophoresis using acrylamide gels. Gel images were then analyzed using FIJI to measure 4R:3R ratio.

### Western blot

Cells were lysed in RIPA lysis buffer (50 mM Tris-Cl pH 8.0, 150 mM NaCl, NP-40, 0.5% sodium deoxycholate, 0.1% SDS) supplemented with 1 mM DTT, 1 mM PMSF, and 1x protease inhibitor cocktail (Roche). Protein concentrations were quantified with the Bio-Rad protein assay kit. 30 μg of total protein was loaded and separated by SDS–PAGE gels. Blotting was performed with standard protocols using PVDF membrane (Bio-Rad). Membranes were blocked for 1 hour in blocking buffer (5% Skim milk in PBST) and probed with primary antibodies at 4°C overnight. After three washes with PBST, the membranes were incubated with HRP-conjugated secondary antibody. Signals were visualized with Clarity Western ECL Substrate (Bio-Rad) as described by the manufacturer. The antibodies used in this assay were: anti-CELF2 (sc-47731, Santa Cruz), anti-GFP (11814460, Roche), anti-tubulin (T9026-2ml, sigma), anti-Histone H3 (A01502, GenScript), anti-HA (ab9110, Abcam), anti-GAPDH (sc-25778, Santa Cruz). For TurboID, the proteins biotinylated by TurboID-tagged CELF2 were detected by Western blot using streptavidin-HRP (016-030-084, Jackson ImmunoResearch).

### Fluorescence imaging

293T or SY5Y cells were seeded on 24-well glass slides (Millipore). Cells were transfected 24 hours after seeding using GFP or mCherry-tag protein construct. 24 hr post transfection, cells were fixed with 4% Paraformaldehyde (Sigma-Aldrich) for 10 min. Then slides were mounted using Prolong Gold Antifade Reagent with DAPI (Life Technologies). Images were captured using a Zeiss microscope (Zeiss LSM780) with a 100x objective. For quantitative analysis, typically, the number of cells displaying GFP-CELF2 foci is determined by counting every 13 GFP-positive cells, and the results are presented as percentages. The fringe visibility of the foci's edges is assessed as amplitude/average value, i.e., (maximum intensity - minimum intensity)/average intensity. FIJI was employed for intensity measurement by drawing a line crossing through the center of the focus and subsequently conducting line measurements. The length of the line is approximately twice the diameter of the focus. 6 representative foci were selected from each cell, and a total of 30 foci were measured.

### Protein interaction assays with LacO array in U2OS 2-6-3 cell line

Human U2OS 2-6-3 cells containing a LacO array with ~50,000 LacO elements in the genome were used to study CELF2-CELF2 homotypic and CELF2-NOVA2/SFPQ heterotypic interactions on chromatin. eYFP-CELF2-LacI plasmids (wt, dIDR, D388A), mCherry-CELF2 plasmids, mCherry-NOVA2 and mCherry-SFPQ plasmids were constructed by the gateway system with our previously constructed eYFP-gw-LacI and mCherry-gw plasmids. For imaging sample preparation, cells were plated on poly-D-lysine-pretreated micro cover glasses (Milipore) on 24-Well Plates (Corning), and were transfected with the target constructs using Lipofectamine 3000 Transfection Reagent (Invitrogen^™^ L3000015) for 24 hours, followed by fixation with 4% paraformaldehyde for 15 minutes. The cells were then mounted with mounting medium containing DAPI. Imaging was performed using Zeiss LSM780 confocal microscope. For quantification, the average signal intensity (I*_center_*) of the mCherry hub was first measured, followed by the measurement of I*_periphery_* (average intensity of four equidistant positions, above, below, left and right of the mCherry hub, with each region having the same area). we calculated the intensity ratio *I_center_*/*I_periphery_* as a measure of the mCherry enrichment at the LacO array. A ratio above 1 suggests protein-protein interactions.

#### Protein purification and in vitro droplet formation assays

Recombinant protein purification was performed as previously described ^83^ with modifications. Constructs used for protein purification were generated through gateway LR recombination between entry clones containing CELF2 cDNA and destination vector pET-His-GFP-gw. *E. coli* Rosetta (DE3) competent cells were transformed with various CELF2 plasmids and protein expression was induced with 0.1 mM IPTG in LB containing 1% glucose. The culture was grown overnight at 18°C and pelleted. The cells were lysed in a Buffer A (50 mM Tris-HCl, pH 7.5, 500 mM NaCl, 10 mM imidazole, protease inhibitor cocktail (Roche)) and subjected to sonication. The total cell lysate was centrifuged at 20,000 rpm for 30 minutes at 4°C, and the soluble fraction was loaded onto a Ni-NTA agarose resin column (Qiagen) that was pre-equilibrated with Buffer A. The resin-bound protein was washed successively with Buffer A and finally eluted with the elution buffer containing a gradient increase in the concentration of Imidazole (50 mM Tris-HCL, pH 7.5, 500 mM NaCl and 50 mM/100 mM/250 mM Imidazole). The eluted proteins were confirmed by running on an SDS Gel, and the proteins were pooled together and subjected to overnight dialysis (50 mM Tris pH 7.5, 125 mM NaCl, 10% glycerol and 1 mM DTT) at 4°C. The dialyzed proteins were concentrated using the 3K centricon (Millipore, Sigma) and proceeded fresh for the following droplet formation assay.

The Purified protein was diluted to 40 μM, 20 μM, 10 μM and 5 μM using the dialysis buffer. The protein was mixed in a 1:1 ratio with droplet formation buffer (50 mM Tris pH 7.5, 125 mM NaCl, 10% glycerol, 1 mM DTT, with 20% or 40% PEG8000) and 5 μl of the suspension was loaded onto a hand-made cassette with a clean glass slide, spacers, and a coverslip. The droplets were immediately observed under an upright confocal microscope (Zeiss LSM780) using a 100x/1.4 oil objective. ZEN black edition version 2.3 was used for acquisition. Droplet size quantification was done using the “analyze particles” tool of FIJI with the threshold set as the medium area of all particles detected in each image.

### OptoDroplet assay

OptoDroplet assay was performed as previously described ^43^ with modifications. OptoDroplet constructs were derived from the pCRY2PHR-mCherryN1 plasmid (Addgene #26866). The plasmid was modified by including a gateway cassette to generate pCRY2PHR-mCherry-gw that is compatible with the gateway cloning system. 48 hours after the transfection of CRY2 fusion constructs into 293T cells that were cultured on coated glass coverslips, cells were transferred to a culture chamber for live cell imaging using a 100x/1.4 oil objective on Zeiss LSM780 microscope. Cells were imaged by using 561 nm of laser for mCherry signal before and 1 min, 5 min, and 15 min after CRY2 activation, which was done using 2% power of 488 nm laser for 30 seconds. Puncta size and number quantification were done using the “analyze particles” tool of FIJI with the threshold set as the medium area of all particles detected in each image.

### CRISPR-Cas13-based RNA imaging

The dCas13b plasmid (3xNLS-dCas13b-3xsfGFP) was a gift from Ling-Ling Chen (Addgene #132404). gRNA plasmids were constructed by cloning gRNA into gRNA vector (Addgene #103854) via the BbsI cloning site. hTaumini-24xGCN4 plasmid was generated by cloning the 24xGCN4 cassette from pmRuby3-24xGCN4 (Addgene #132413) to Pcmv-hTaumini. 0.1 μg of dCas13b-GFP, 0.2 μg of gGCN4-1, 0.2 μg of gGCN4-2 (or 0.4 μg of gNC), 0.1 μg of hTaumini-24xGCN4, and 0.1 μg of mCherry-CELF2 were co-transfected to HEK293T cells cultured on coverslips in 24-well plates using lipofectamine 3000. 48 hours post transfection, live cells were imaged using a 100x lens and the Zeiss LSM780 microscope.

### KEGG pathway analysis of CELF2 targets

The CLIP-seq data was obtained from our previous publication ^35^. After the reads were trimmed and cleaned, they were aligned to mouse genome (mm9) with STAR 2.5.2b ^84^. Only uniquely mapped and without-duplication reads were selected for subsequent analysis. Piranha was used to identify regions of statistically significant read enrichment, with bin_size set to 500bp ^85^. ChIPseeker was used to annotate and associate the peaks with genes ^86^. The whole cell lysate CLIP-seq data was used to identify the potential target genes (4279 genes), which were then subjected to DAVID (https://david.ncifcrf.gov/) KEGG pathway analysis.

### TurboID and proteomics data analysis

The pRetroX-TurboID-HA was obtained from Addgene (plasmid #36047). The plasmid was modified by including a gateway cassette to generate pRetroX-TurboID-HA-gw that is compatible with the gateway cloning system. The human CELF2 cDNA was then cloned into pRetroX-TurboID-HA-gw using the Gateway system to get pRetroX-TurboID-HA-Celf2. The pRetroX-TurboID-HA-Celf2 was co-transfected with pCL-Ampho packaging plasmid into 293T cell line to produce retrovirus. The retrovirus was then transduced into a parental SH-SY5Y cell line that was engineered to stably express a Tet Repressor using a retroviral vector. To induce TurboID-HA-Celf2 protein expression, 1 μg/ml doxycycline was added into culture media approximately 24 hours before adding 50 mM Biotin for another 15 minutes to label all proteins in the proximity of CELF2 before collection.

To detect CELF2-associated nuclear proteins, nuclear lysate fraction was incubated with 200 μl PBS-washed MyOne Streptavidin T1 Dynabeads (Life Technologies) with overnight rotation at 4°C to pull down biotinylated proteins. The beads were then washed for five times with RIPA buffer with 10 min rotation for each wash. After 5X washes with RIPA, the beads were washed 2X with stringent RIPA buffer containing 5% SDS, followed by 4X washes with PBS. 10% of the beads suspension was used for western blot and 90% was used for mass spectrometry. For western blot, the beads were boiled for 10 min in 2X protein loading buffer with 5% beta-Me to release proteins. For mass spectrometry, beads were stored at −80°C before shipping to the IDeA National Resource for Quantitative Proteomics.

MS raw files were analyzed by MaxQuant software with default settings, and Andromeda search engine was used to search against the human Uniprot database. False discovery rate was set to 0.01 for proteins and peptides with a minimum length of seven amino acids. Variable modifications were set to methionine oxidation and N-terminal acetylation; while fixed modification was set to carbamidomethylation. For label-free protein quantification, the minimum ratio count was set to two, and peptides for quantification were set to unique and razor. Statistical analysis was performed by Perseus. Proteins identified only by site, reverse hits or potential contaminants were removed before downstream analysis. LFQ intensities were log2 transformed, and the matrix was then grouped with regard to cell lines. The proteins were then filtered by requiring at least two valid values in at least one cell line. Missing values were imputed based on normal distribution. Paired t-test was then used to determine the significant difference of protein level between the two cell lines.

#### Generation of transgenic C. elegans strains

*C. elegans* were maintained on nematode growth medium (NGM) agar plates with Escherichia coli strain OP50 at 20°C using standard procedures unless stated otherwise. Transgenic *C. elegans* strains were generated by microinjecting plasmid DNA into the syncytial germline of young adult hermaphrodites. The expression construct was injected at 5–10 ng/μL (5 ng/μL for CELF2 constructs and 10 ng/μL for tau constructs) together with a co-injection marker ttx-3-rfp at 90 ng/μL. Microinjections were performed using standard differential interference contrast (DIC) microscopy and pulled borosilicate needles. Injected animals were recovered on seeded NGM plates and allowed to self-fertilize. F1 progeny expressing the co-injection marker were selected to establish stable extrachromosomal lines.

### Crawling velocity quantification

Crawling locomotion was assessed using video recording coupled to ImageJ analysis. Day-1 adult worms were transferred to fresh, bacteria-free plates and allowed to acclimate for 1–2 min to minimize handling-induced behavioral artifacts. Videos were recorded using a digital camera mounted on a stereomicroscope at 1x magnification with a frame rate of 15 frames/s for 20 sec. Video files were imported into ImageJ (FIJI distribution) and converted to 8-bit grayscale. Crawling speed was measured by tracking the worm’s nose tip or center of mass across frames with the wrMTrck plugin. At least 15–20 worms per condition were analyzed in blinded fashion.

#### Gentle touch assay in C. elegans

Gentle touch responses were assessed using established methods to evaluate the function of the six mechanosensory touch receptor neurons. Day-1 adult hermaphrodites were transferred to NGM plates and allowed to acclimate for 1–2 minutes before testing. Animals were gently stroked on the anterior or posterior body region using an eyelash glued to a toothpick, ensuring minimal displacement of the worm and avoiding direct pressure. For each worm, five anterior and five posterior touches were delivered in alternating order with ~ 10 seconds between stimuli. A positive response to anterior touch was scored as reversal, whereas a positive response to posterior touch was scored as forward acceleration. Animals that paused, made incomplete movements, or exhibited spontaneous locomotion changes during stimulation were scored as non-responsive. Touch assays were performed blinded to genotype, and data were quantified as the percentage of correct responses out of total stimuli delivered.

### Chemotaxis assay

Chemotaxis assay to assess olfactory-dependent chemotaxis and memory-like behavior was performed as previously described ^87^. For conditioning, Day-1 adult worms were transferred to unseeded “conditioning” plates and exposed to vapors of Isoamyl alcohol (IA, Sigma I9392) in the absence of food, to associate IA with starvation. For the chemotaxis assay, large NGM plates (no OP50) were marked with three zones: a start point (“S”), an IA source (“IA”), and a trap/control spot (“T”) forming an isosceles-triangle layout on the agar surface. Fifteen minutes before worm loading, 15 μL of 20 mM sodium azide was added to the “IA” and “T” spots to immobilize worms arriving there. Worms were washed gently from conditioning plates using M9 buffer and loaded onto the “S” spot of the assay plate using glass pipettes; excess buffer was removed with a delicate-swipe filter paper to minimize stress. A small square (~ 0.5 × 0.5 cm) of parafilm was placed at the “IA” spot, and 4 μL of 1:50 diluted IA in water was applied onto the parafilm to allow slow diffusion. Plates were immediately sealed and incubated at 20°C for 2 h. After incubation, the number of worms in each zone (“IA”, “T”, or remaining in “S”) was counted under a microscope. The chemotaxis index or memory-related behavior score was calculated as (N_T – N_IA) / N_total, where N_T and N_IA are the numbers of worms at the trap and IA zones, respectively. All assays were performed in parallel for control and experimental groups, and scoring was done blinded to genotype or treatment.

### Statistical analysis

Each experiment was repeated at least twice to make sure that results are reproducible. Results are representative of at least three biological replicates and reported as mean ± SEM or mean ± SD. Data were analyzed and statistics were performed using unpaired two-tailed Student’s *t*-tests or one-way ANOVA (Prism 5 GraphPad). significant differences between two groups were noted by asterisks (* P < 0.05, ** P < 0.01, *** P < 0.001).

## Supplementary Material

This is a list of supplementary files associated with this preprint. Click to download.

• supfiguresreducedsize.pdf
